# Understanding Key Predictors of Life Satisfaction in a Nationally Representative Sample of Koreans

**DOI:** 10.3390/ijerph20186745

**Published:** 2023-09-12

**Authors:** Yun-Kyeung Choi, Mohsen Joshanloo, Jae-Ho Lee, Hong-Seock Lee, Heung-Pyo Lee, Jonghwan Song

**Affiliations:** 1 Department of Psychology, Keimyung University, Daegu 42601, Republic of Korea; ykchoi@kmu.ac.kr (Y.-K.C.);; 2 Hallym University Medical Center, Seoul 07441, Republic of Korea; 3 Department of Art Therapy, Daegu Cyber University, Gyeongsan-si 38453, Republic of Korea

**Keywords:** life satisfaction, well-being, gender, age, income, Gallup World Poll

## Abstract

The purpose of this study was to examine the factors that predict life satisfaction in a large representative sample of Koreans by analyzing data from the Gallup World Poll. The primary objective was to identify important predictors and suggest strategies to improve quality of life in Korea. The study used available Korean data from 2006 to 2017, which included 14,101 participants (mean age = 46.42). Predictors included demographic and psychological variables, with the Cantril Ladder of Life Scale serving as the outcome variable. The results show a decline in life satisfaction with advancing age, and that the relationship between life satisfaction and age varied by gender. Among the predictors examined, satisfaction with the standard of living and household income emerged as the most influential factors in determining life evaluation; other strong predictors included positive affect and negative affect, social support, gender, and education level. These results imply that, to increase life satisfaction, it is imperative to provide job opportunities and social services specifically targeted to individuals in low-income groups. In addition, it is crucial to implement tailored psychosocial interventions that address the unique developmental tasks and psychological challenges experienced by individuals according to their gender and life cycle stage.

## 1. Introduction

### 1.1. Introduction

Advances in science, technology, and social progress have brought about a profound societal change, shifting our focus from the mere pursuit of longevity to a deeper understanding of how to live a fulfilling existence. This shift is reflected in the growing emphasis on the study of quality of life and mental well-being. Quality of life research encompasses the holistic well-being of both individuals and society, shedding light on the positive and negative aspects of our lives [1]. Early studies in quality-of-life research relied on economic indicators such as gross national product (GNP) to assess national well-being [2]. Since the 1960s, however, attempts have been made to measure quality of life using objective social indicators, including educational attainment, political participation, population growth, and migration patterns [3]. Since the 1980s, there has been a consistent practice of conducting regular surveys on social indicators that have contributed greatly to our understanding of how people’s living conditions have evolved over time. These surveys provide valuable insights into the changes that have occurred in various aspects of people’s lives [4].

However, these social indicators have limitations in directly capturing the quality of life experienced by individuals as perceived by themselves. In other words, although objective environmental conditions such as social or economic indicators influence quality of life, they cannot be equated with quality of life itself as subjectively experienced by individuals [4]. Consequently, psychologists have emphasized subjective well-being as a key indicator of quality of life, which directly captures subjective experiences. Some psychologists recognize that subjective well-being and life satisfaction are more important to individuals than objective indicators. Life satisfaction, one of the indicators of a fulfilling life sought by individuals, represents an individual’s cognitive evaluation of his or her overall life [5]. High life satisfaction is associated with extraversion [6], self-esteem, positive interpersonal relationships [7], optimism, low depression [8], positive emotions [7,8], and emotional stability [6]. Life satisfaction is considered a valuable indicator for understanding individual adjustment and well-being and is worthy of further study in various cultural contexts.

Sociocultural and historical events shape cohorts in different ways, resulting in differences in the values they pursue and the conditions they consider essential for life satisfaction. In addition, perceptions of life satisfaction are influenced by sociodemographic factors such as gender, marital status, and age and psychosocial factors such as emotional experience and social support within an individual’s social and cultural context. In societies characterized by instability, such as income inequality, gender inequality, and intergenerational conflict, factors such as financial stability and social security become particularly important for individuals’ well-being [9,10,11,12].

Following the period of Japanese colonial rule (1910–1945) and the Korean War (1950–1953), Korea has emerged as a nation with rapid economic growth and remarkable industrialization in a relatively short period of time. However, this rapid development and growth have come with negative consequences, including income polarization, high youth unemployment, the emergence of an aging society, and the unfortunate distinction of having the highest suicide rate among OECD countries [13].

According to the World Happiness Report, Korea ranked 41st out of 156 countries with a score of 6.27 in the 2013 report, 47th out of 158 countries with a score of 5.984 in the 2015 report, and 57th out of 156 countries with a score of 5.875 in the 2018 report [14,15,16]. The latest ranking of Korea was 57th out of 137 countries with a score of 5.951 in the 2023 report [17]. Korea consistently ranks in the 50s and 60s among some 150 countries, with an average score below 6. Thus, despite its top 10 global economic ranking [18], the happiness of its population does not reach comparable heights.

As many developing countries seek economic progress and look to Korea as a potential model, they must consider key aspects such as life satisfaction in the Korean context. Therefore, it is important to conduct comprehensive research on the factors that influence the life satisfaction of Koreans. There is a lack of recent, comprehensive research on the predictors of subjective well-being using nationally representative Korean samples. Our study aimed to fill this gap.

### 1.2. Sociodemographic Factors and Life Satisfaction

It is widely accepted that various sociodemographic and psychological factors influence life satisfaction [10,19,20,21,22,23]. Among the predictors of quality of life, income is typically considered because it meets basic survival needs, influences freedom from poverty, and provides material wealth in modern society [9,24]. Many studies have consistently shown that material wealth alone does not guarantee mental well-being [20]. In this regard, subjective satisfaction with household income and standard of living is more important than actual income levels [19].

According to the income rank hypothesis, an individual’s life satisfaction is predicted by their rank position in terms of income, rather than their absolute or reference income [12,25]. Furthermore, the association between individual income and life satisfaction is influenced by income inequality in studies conducted at the country level [12]. A meta-analysis revealed that the overall correlation between income inequality and life satisfaction was not statistically significant. However, this association was found to be influenced by the economic development of the country [26]. Yet, a recent study using large UK panel data showed that rank, household income, and reference income were all important explanatory variables for life satisfaction [27]. Subjective poverty was found to be an important predictor of the life satisfaction of European adults over the age of 50, as determined by a machine learning approach [28]. In addition, studies have shown that the relationship between income inequality and life satisfaction is complex. This complexity arises from a phenomenon known as the “tunnel effect”, where income inequality can actually enhance well-being by serving as a signal to lower earners that their own circumstances may be improving [12]. These complex and mixed findings suggest that more empirical research is needed to clarify the relationship between income and life satisfaction, and the relationship depends on the sociocultural context in which it is investigated. In addition, moderators such as gender and age should be considered. An important aspect of the relationship between income and well-being is its non-linear nature. In many countries, the relationship between income levels and life satisfaction reveals a steep increase in satisfaction up to a certain income threshold, followed by a plateau above that threshold [29]. The specific income threshold varies among countries, but the observed trend is consistent across all of them.

Differences in life satisfaction across countries are mainly due to differences in sociocultural and political contexts, resulting in different patterns of life satisfaction in relation to sociodemographic variables, as well as different associations of life satisfaction with gender and age. For example, differences in life satisfaction by gender and age were investigated in countries such as Japan [30], Serbia [31], Australia [32], United Arab Emirates [33], Malaysia [34], Italy [35], and New Zealand [36], and it was confirmed that high life satisfaction was associated with higher levels of education [30,31,33,34], income [31,32,34,36], satisfaction with standard of living [30,31,32,34,35,36], and household income satisfaction [30,31,32,34,35,36].

Many studies have reported a U-shaped relationship between age and life satisfaction, with a minimum around middle age [37]. However, it has been argued that this U-shaped pattern is mostly prevalent in some high-income (e.g., English-speaking) countries [38]. Subsequent research has produced mixed results, including a decline in life satisfaction with age [39] or even an S-shaped pattern with a second decline occurring after the age of 70 [1]. The relationship between age and life satisfaction has also shown different trends by gender (e.g., [34]).

A study analyzing multidimensional poverty, which includes income, schooling, health, and social protection, discovered that China has a higher prevalence of multidimensional poverty compared to Japan or Korea. However, the study also revealed that the largest gender and age differences in multidimensional poverty are observed in Korea [11]. Korea’s relative poverty rate in 2020, according to the Statistics Research Institute (2023, [40]), was 15.3%. This rate is slightly higher than that of Australia (12.6%), England (11.2%), Germany (10.9%), and France (8.4%) among OECD countries. However, the relative poverty rate for the population aged 65 and over in Korea was 40.4%, significantly higher than that of Costa Rica (22.4%), Mexico (19.8%), and Japan (20.0%), all of which are countries with high relative poverty rates. Against the backdrop of Korea’s socioeconomic and cultural context, it is pertinent to examine whether the determinants of life satisfaction among Koreans show similarities or differences compared to those in other countries. Furthermore, gender and age can act as moderating variables in predicting life satisfaction, further highlighting the complex interplay between different predictors (e.g., [30]). It is therefore important to determine whether the relationship between income and life satisfaction differs by gender and age.

### 1.3. Psychosocial Factors and Life Satisfaction

Numerous psychosocial variables significantly contribute to an individual’s life satisfaction. The following are the psychosocial factors that have received attention in the research literature for their influence on life satisfaction: the pursuit of a meaningful life, the experience of positive and negative emotions [21,30,31,32,35], perceived social support [31,34,35,41,42,43], self-perceived health [28], freedom [30,31,32], safety at night [30,36], satisfaction with the city [30,31,32,36], and respect received [30]. Notably, in one study, perceived social support, along with income level, was the most consistent predictor of overall and domain-specific life satisfaction [43]. Given that life satisfaction can be primarily achieved through personal success and self-esteem in individualistic cultures like North America, and through supportive social interactions in collective cultures like East Asia [44], perceived social support may play a significant role as a key predictor of life satisfaction in Korea.

### 1.4. Current Study

The purpose of this study was to comprehensively analyze the predictors of the quality of life among Koreans, with a focus on life satisfaction as a subjective indicator. We aimed to investigate the influence of important sociodemographic and psychosocial factors, including age, gender, satisfaction with standard of living, household income satisfaction, emotional well-being, and social support. Specifically, our research questions were as follows: (1) How are sociodemographic factors such as income, age, and gender associated with life satisfaction in Korea? (2) What psychosocial factors explain the age and gender disparities in life satisfaction in Korea? (3) What sociodemographic and psychosocial factors are important across different age, gender, and income groups in Korea? (4) What is the nature of the relationship between annual household income and life satisfaction in Korea? To identify the key predictors that significantly influence life satisfaction, we utilized a large and nationally representative sample of Koreans from the Gallup World Poll.

## 2. Methods

### 2.1. Participants

Data for this study come from the Gallup World Poll, which has been collecting nationally representative samples from Korea since 2006. Each year, individuals in Korea aged 15 and older are contacted by landline and/or cell phone to participate in the survey. Interviews are about 30 min. In countries where telephone surveys are conducted, including Korea, Gallup uses either random digit dialing or a nationally representative list of telephone numbers. The survey team makes at least three attempts to contact an individual in each household. To ensure the random selection of respondents, Gallup uses either the latest birthday method or the Kish grid method (https://news.gallup.com/poll/105226/world-poll-methodology.aspx, accessed on 1 July 2023). No incentives were given to participants. The sample size for most years is approximately 1000, with the exception of 2012 and 2014, which have sample sizes of 2000. For our analyses, we used all available Korean data from 2006 to 2017, resulting in a total of 14,101 participants. No participant was excluded from the analyses.

### 2.2. Measures

For our study, we used a selection of items from the Gallup World Poll collection that previous research has identified as relevant predictors of mental well-being (e.g., [19]). These items, along with their response formats, are presented in Table 1. Among the measures included in our analysis was the Cantril Ladder of Life Scale [45], which assesses life satisfaction and serves as the outcome variable for this study.

### 2.3. Data Analysis

Given the weak intercorrelations observed between the items, each item was used as a separate variable. However, based on the results of separate factor analyses, three composite variables were computed and used. The results of the principal axis factor analysis indicated that stress, worry, sadness, and anger together formed a single factor (Eigenvalue = 2.22, explaining 55.57% of the variance), with factor loadings ranging from 0.56 to 0.73 (α = 0.73). The first Eigenvalue was 2.22, whereas the remaining Eigenvalues were smaller than 0.80, which is consistent with a single-factor structure. In addition, laughter and enjoyment were found to form a factor (Eigenvalue = 1.55, explaining 77.80% of the variance), with factor loadings of 0.745 (α = 0.71). Finally, perceptions of business and government corruption were also found to constitute a single factor (Eigenvalue = 1.55, explaining 77.58% of the variance), with a factor loading of 0.742 (α = 0.71).

Descriptive statistics and data visualization techniques were used to examine the distribution of life satisfaction across demographic groups. T-tests were used to assess gender differences. An analysis of variance (ANOVA) was used to examine the association between categorical predictors and life satisfaction. In addition, multiple regression analyses were used to examine the relationships between continuous and binary predictors and life satisfaction, both in the overall sample and separately for different demographic groups.

## 3. Results

### 3.1. Sociodemographic Characteristics of the Sample

**Figure 1:**
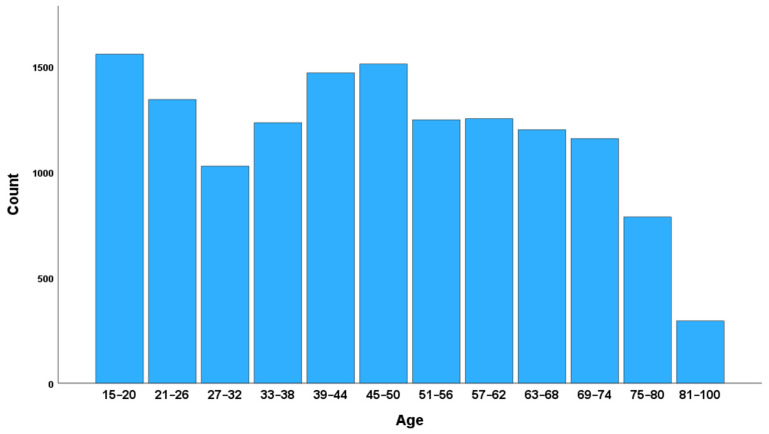
Age distribution.

The sample consisted of 14,101 participants (50.2% female, M_age_ = 46.42, SD_age_ = 19.08). The age distribution is shown in Figure 1. Of the total sample, 30.4% reported to be Christians, 15.8% reported to be Buddhists, 36.5% reported to have no religion, and 2.4% reported other religions (14.9% missing data). Of the total sample, 21.1% reported their employment status to be “Employed full time for an employer”, 9% chose “Employed full time for self”, 5.7% reported “Employed part-time do not want full time”, 2.9% reported to be unemployed, 2.9% reported “Employed part-time want full time”, and 36% reported being out of the workforce (22% missing data). Of the total sample, 11% completed elementary education or less (up to 8 years of basic education), 53% completed secondary education or some education beyond secondary education (9–15 years of education), 35% completed four years of education beyond high school and/or received a 4-year college degree, and less than 1% of the participants reported that they did not know their education level or refused to answer.

### 3.2. How Does Life Satisfaction Vary in Korea by Age and Gender?

**Figure 2:**
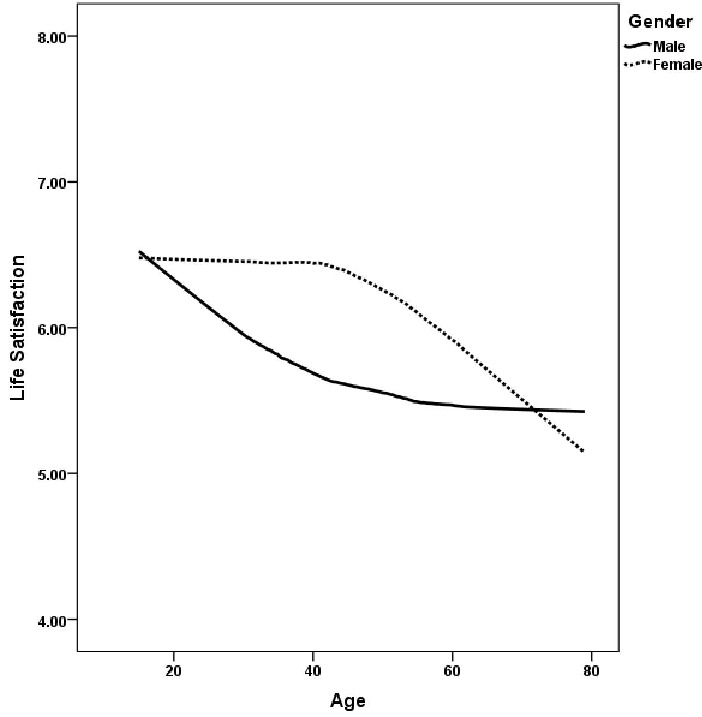
Life satisfaction by age and gender.

A *t*-test analysis revealed significant differences between men and women in life satisfaction, with women scoring significantly higher than men [*t*(13,057.021) = −14.033, *p* < 0.001, 95% CI of difference: −0.665, −0.502, *d* = 0.245], indicating a small effect size. Figure 2 shows the distribution of life satisfaction by age and gender, with locally weighted smoothing (LOESS) applied to the scatterplots to improve data presentation. Individuals over the age of 80 have been excluded from the graph due to the relatively small sample size. The graph shows that women’s life satisfaction remains relatively stable from age 15 to about age 40, after which it gradually declines. In contrast, men experience a steady decline in life satisfaction from about age 15 to age 50, followed by a relatively stable pattern. These findings suggest a general decline in life satisfaction with advancing age in South Korea. In addition, women report higher levels of life satisfaction than men between the ages of 20 and 60. Notably, the observed increase in life satisfaction after the age of 60, which is commonly observed in some countries [46], was not evident in the Korean sample.

As a supplementary analysis, we also looked at gender differences in affect. The results of *t*-test analyses showed that women scored significantly higher on negative affect [*t*(13,088.444) = −2.920, *p* = 0.004, 95% CI of difference: −0.026–0.005, *d* = 0.050] and positive affect [*t*(13,025.703) = −9.782, *p* < 0.001, 95% CI of difference: −0.085–0.057, *d* = 0.171] than males, yet the effect sizes were small, particularly for negative affect.

### 3.3. Explaining Age and Gender Differences in Life Satisfaction

**Figure 3:**
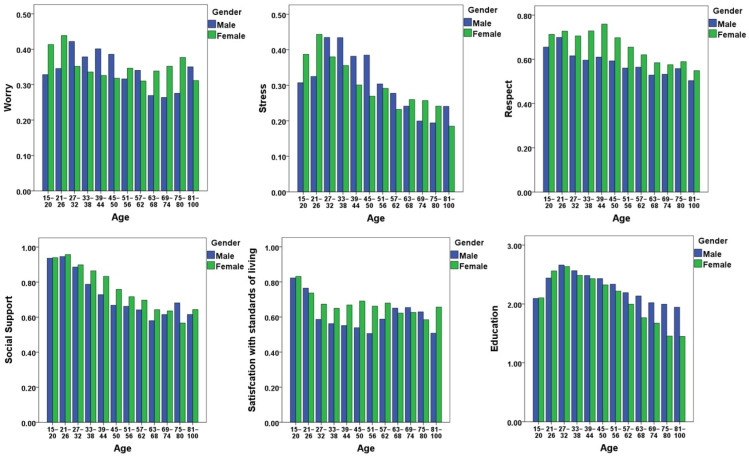
Age and gender patterns in predictors of life satisfaction.

To elucidate the underlying factors that contribute to the observed demographic patterns in life satisfaction in Korea, we examined the age and gender patterns in various variables within the data set. Figure 3 shows the distributions of six variables by age and gender. Differences in these variables by age and gender partially explain the observed differences in life satisfaction. In particular, women have higher levels of life satisfaction than men between the ages of 20 and 60. The graphs show that, on average, women report lower levels of worry and stress than men before the age of 60. Conversely, before the age of 60, women report higher levels of perceived social support, respect received, and subjective satisfaction with living standards than men. However, women’s advantages in these variables diminish after the age of 60. In addition, older Korean women tend to have lower levels of education than older men, which may contribute in part to the notable decline in well-being among older Korean women.

### 3.4. Other Demographic Predictors of Life Satisfaction

Table 2 presents the results of five different ANOVAs using the demographic variables as independent variables to explain variations in life satisfaction. For religious affiliation and relationship status, certain categories with particularly small sample sizes (e.g., “Hindu” and “domestic partner”) were either excluded or combined into an “other” category. Education level emerged as the most influential predictor of life satisfaction, accounting for 4.4% of the variance, followed by relationship status, which accounted for 1.5% of the variance. Specifically, individuals with higher levels of education reported significantly greater satisfaction than those with lower levels of education. Notably, there was no significant difference in life satisfaction between single and married participants. However, individuals who were widowed reported lower life satisfaction compared to both married and single participants. An additional ANOVA indicated that gender did not moderate the relationship between relationship status and life satisfaction. Employment status, religious affiliation, and location together explained 1% or less of the variance in life satisfaction. An overall ANOVA including all five variables showed that these variables together accounted for approximately 6.2% of the variance in life satisfaction (with a moderate effect size), with education and relationship status emerging as the most significant predictors. When assessed simultaneously, the unique contributions of each variable were reduced to 0.9%, 2.5%, 0.1%, 0.7%, and 0.1% for employment, education, location, religious affiliation, and relationship status, respectively.

### 3.5. Comprehensive Prediction of Life Satisfaction

A regression analysis was conducted using the ENTER method in SPSS, including all predictors of life satisfaction as well as relevant demographic variables. The results are shown in Table 3. The analysis included a total sample of 6784 participants with complete data on all 28 variables. The combined set of predictors accounted for approximately 31% of the variance in life satisfaction scores, as indicated by an *F*-test (*F*(27, 6756) = 111.027, *p* < 0.001, *R*^2^ = 0.307). This is indicative of a large collective effect size. Notably, 9 of the 27 predictors did not emerge as significant predictors of life satisfaction at the 0.05 significance level. A separate stepwise regression analysis identified satisfaction with living standards as the most influential predictor, explaining approximately 17.2% of the variance. Satisfaction with household income emerged as the second strongest predictor, contributing an additional 5% of the explained variance. Positive affect, social support, and education followed as important predictors, explaining 2.5%, 1.4%, and 1.1% of the variance, respectively. Together, these five variables accounted for 27.2% of the variance in life satisfaction scores. The remaining variables together contributed an additional 3.4% of the explained variance.

The stepwise regression results indicated that age squared, giving behavior, feeling safe at night, living in a rural area or small town, employment status (full time and “part-time, not looking for full-time”), being out of the workforce, and identifying as a Buddhist were the least important predictors and were therefore excluded from the regression equation. In addition, separate regression analyses were conducted across age and gender groups using the ENTER procedure. The results presented in Table 4 and Table 5 also include the five most significant predictors for each group, obtained through separate regression analyses using the stepwise procedure. Following the work of [47], four age categories were used to represent emerging adulthood (15–25), young adulthood (26–44), middle adulthood (45–64), and late adulthood (65 and older). This categorization was inspired by previous studies by Erikson [48] and Arnett [49]. The variable “widow” was excluded as a regressor in the analyses across age groups due to either a lack of cases or very small sample sizes in the younger age categories. The results indicated that the regression coefficients were generally similar across gender groups in terms of the percentage of variance explained and the order and significance of the predictors. However, greater discrepancies in regression coefficients and explained variance were observed between the different age groups.

### 3.6. Relationship between Household Income and Life Satisfaction: A Close Examination

Table 6 shows the levels of life satisfaction corresponding to each household income quintile. An ANOVA was conducted using income level as a predictor of life satisfaction. The results showed a significant prediction of life satisfaction by income level (*F*(4, 10,194) = 199.685, *p* < 0.001, *η*^2^ = 0.073), accounting for 7.3% of the variance (indicating a moderate effect size). In addition, a separate ANOVA revealed that the influence of income on life satisfaction was not affected by gender.

**Figure 4:**
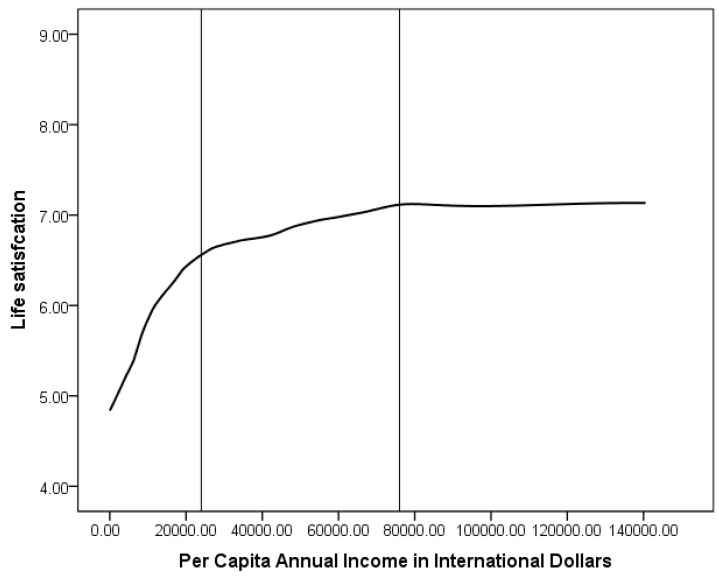
The relationship between annual household income (in international dollars) and life satisfaction in Korea. Note. The vertical lines mark approximate boundaries between low-, medium-, and high-income groups.

The relationship between annual household income per capita (in international dollars) and life satisfaction is shown in Figure 4. Individuals with incomes above 150,000 international dollars were excluded due to the small sample size. As can be seen, the relationship between income and life satisfaction is relatively strong in the low-income group (less than 24,000 international dollars, equivalent to about KRW 22,000,000). In the middle-income group (24,000–76,000 international dollars, equivalent to about KRW 22,000,000–68,000,000), the relationship is less strong. Finally, in the high-income group (over 76,000 international dollars or KRW 68,000,000), the relationship is almost flat. However, the patterns associated with the last category should be interpreted with caution, as the sample size at the upper end of the income distribution is extremely small.

We also looked at the predictors of life satisfaction across the different groups of income quintiles. The results are shown in Table 5 and Table 7. Table 5 shows the list of top predictors for each income group.

## 4. Discussion

Increasing the level of subjective well-being among Koreans is one of the challenges facing Korean society. The purpose of this study was to identify important predictors among sociodemographic and psychosocial factors of life satisfaction among Koreans and to examine the social implications of the findings. The results reveal several noteworthy observations regarding the effects of sociodemographic and psychosocial factors on life satisfaction. First, women reported higher life satisfaction than men, although the effect size was small. The analysis revealed significant gender differences in the relationship between age and life satisfaction, with characteristics of a slightly U-shaped curve for men and an inverted U-shaped curve for women. The gender difference in the relationship between age and life satisfaction showed an increasing trend until middle age, followed by a decline. These patterns reflect the complex social and psychological context of Korea. An important factor to consider is the influence of gender inequality within families, especially among older cohorts [9,11]. Older women tend to have lower levels of educational attainment than men [50]. Educational attainment plays a critical role in providing multiple pathways to success in Korean society and serves as a significant predictor of life satisfaction [51]. In this study, education level had a significant effect on life satisfaction. In addition, before the age of 60, women had lower levels of worry and stress and higher scores on social support, respect, and satisfaction with living standards than men. In addition, Korean men in young and middle adulthood face a number of developmental challenges, including military service, employment, work–life balance, marriage, and the financial responsibilities associated with supporting their families [52]. These cumulative psychological stresses may contribute to a decline in life satisfaction, especially in middle age. Due to rapid modernization, Korean women now face many of the same challenges as men, with the notable exception of military service.

Second, the life satisfaction of Koreans showed a declining trend with increasing age. This decline in life satisfaction among individuals as they age suggests the influence of our social culture in recent decades. The growing elderly population, coupled with inadequate measures to address their needs, is a matter of great concern in Korean society. Various factors, such as a decline in household income among elderly households [53], an increase in economically vulnerable single-person households [54], and the absence of a well-developed leisure culture tailored to the elderly [53], may negatively affect the quality of life of older adults. As a result, there is a potential deterioration in the overall well-being of the older population.

Similar patterns of decreasing life satisfaction with age have been observed in countries such as Japan [30], Serbia [31], Malaysia [34], and Italy [35], while New Zealand showed an opposite pattern of increasing life satisfaction with age ([36]). Blanchflower and Oswald [37], in an analysis of data from about 80 countries, reported a curvilinear relationship between well-being and age, with happiness following a U-shaped pattern across the life course, reaching a low point in middle age. However, the U-shaped relationship between happiness and age is primarily observed in some high-income countries, while declines in happiness with age have been reported in the former Soviet Union, Eastern Europe, and Latin America, and little change in happiness across different ages in sub-Saharan Africa [39]. Studies using the same Gallup survey data have found U-shaped curves in the relationship between age and life satisfaction in Australia [32], New Zealand [36], and the United Arab Emirates [33]. Notably, the relationship between age and life satisfaction differs by gender in Korea.

Third, the remaining five demographic variables—education, relationship status, employment, religious affiliation, and location—excluding gender and age, contributed to life satisfaction with effect sizes ranging from small to medium. In particular, the level of education and relationship status, especially widowhood, emerged as significant factors, although the effect size remained small. As mentioned earlier, education plays a pivotal role in shaping life opportunities in Korean society [51]. While marital status (married or single) did not show a difference, being widowed was associated with lower life satisfaction. The loss of a spouse is considered one of the most stressful life events across all cultures. The experience of losing a loved one can elicit negative emotions, such as sadness and depression, and lead to a decrease in social support, ultimately affecting overall life satisfaction [55,56].

Fourth, the comprehensive regression model with 27 predictors significantly accounted for life satisfaction and showed a large overall effect size (i.e., *R*^2^). Using only five key variables—satisfaction with standard of living, satisfaction with household income, positive affect, social support, and education—the model remained significant again with a large effect size. These findings are consistent with previous research (e.g., [31,35]). Satisfaction with the standard of living had a medium to large effect, satisfaction with household income had a small to medium effect, and positive affect had a relatively modest effect. The effects of social support and education were relatively weaker. When the full sample was disaggregated by gender, age group, and income quintile, the main predictors within each subgroup had consistently large effects on life satisfaction. In addition, there are no significant differences in the predictors of life satisfaction by gender, but there are slight differences by age group and income quintile. In particular, satisfaction with the standard of living, positive affect, and satisfaction with household income stand out as the three key predictors in almost all subsamples.

Looking at the characteristic predictors in each subsample, freedom and learning experiences emerged as significant factors in the 15–24 age group. Social support emerged as an important predictor of life satisfaction among middle-aged and older adults, as well as among low-income adults. Kim et al. [57] also found that satisfaction with family (marital life), a facet related to social support, had the greatest weight as a determinant of happiness. In addition, respect and helping others were important predictors of life satisfaction in the fourth income quintile group, while negative emotions and satisfaction with the city were identified as important predictors in the wealthiest quintile group. Education was a more important predictor for middle and late adulthood than for adolescence and young adulthood. This finding is consistent with previous research. For example, in South Korea and Japan, where higher education is widely accessible, educational attainment was not found to be a significant factor in life satisfaction among younger age groups [22,30]. Conversely, educational attainment emerged as a notable predictor of life satisfaction among the middle-aged and older cohorts [9,11], suggesting the importance of educational opportunities in shaping socioeconomic status and quality of life in adulthood [58].

Finally, the relationship between annual household income and life satisfaction in Korea shows a graph pattern consistent with previous studies. Particularly striking is the robust relationship observed between household income and life satisfaction among low-income households, while this relationship tends to weaken once household income exceeds a certain threshold. This pattern is consistent with findings from studies conducted in other countries [31,32,34,36] and underscores the notion that the positive impact of income growth on life satisfaction is more pronounced among individuals with lower income levels. However, once household income exceeds a certain threshold, the impact of income on life satisfaction diminishes [31,32,34,36], suggesting that factors beyond income play an increasingly important role in shaping overall life satisfaction. Beyond a certain income threshold, non-income factors become increasingly important in determining an individual’s overall life satisfaction.

Based on these findings, several measures can be considered to improve the quality of life for Koreans. First, it is crucial to address social issues such as income inequality and polarization, as household income has been identified as a significant determinant of life satisfaction [59]. It is imperative to implement social support programs and welfare initiatives, including financial assistance for low-income individuals to meet their basic living needs [60]. In particular, ensuring employment opportunities with secure income is essential for improving life satisfaction. Stable employment and the ability to work are fundamental to life satisfaction [9,30,32,36,60,61]. It is critical to recognize that beyond income, the fulfillment of personal aspirations and the opportunity to engage in meaningful work can significantly contribute to life satisfaction [62]. Through work, individuals can experience a sense of accomplishment, cultivate self-efficacy, and increase overall life satisfaction [62,63].

Second, it is imperative to explore the predictors of life satisfaction and gain insight into the developmental tasks associated with different genders and life stages. Factors such as employment, marriage, retirement, and coping with the loss of a loved one can significantly affect life satisfaction [60]. It is particularly important to understand the specific developmental tasks that are unique to each gender and life stage within the sociocultural context of Korea. Such an understanding can provide valuable insights to guide interventions and strategies aimed at enhancing life satisfaction.

Third, Korean society is currently experiencing rapid population aging, leading to concerns about potential population decline. Korea has the highest elderly poverty rate among the 20 OECD countries, according to 2018 data [64]. In addition, income inequality often persists after retirement due to the lack of a fixed income for several years. Middle-aged people express concern about their lives in old age, but practical and concrete preparations are insufficiently implemented [65]. The elderly face the reality of living on a limited income and must adjust their lifestyles accordingly. The alarmingly high rate of elderly suicides in Korea requires immediate attention. Specifically, as of 2016, the suicide rate (per 100,000 population) for people aged 65 and older was 53.3, and by 2020, it had declined to 41.7. Nevertheless, these rates remain significantly higher than the average rates observed in OECD member countries [13]. In an aging society, targeted social and psychological interventions are crucial for improving the quality of life of the elderly.

Therefore, it is essential to focus efforts on implementing policies and interventions that effectively address the socioeconomic challenges and mental health crises experienced by older people. This includes measures to ensure their financial security and the establishment of comprehensive support systems aimed at preventing social isolation and improving psychological well-being [9,60,66]. Adequate preparation for old age should begin at least in middle age, and psychoeducational initiatives, including economic education, can play a key role in promoting a high quality of life. In addition, it is crucial to identify the factors that contribute to the gradual decline in life satisfaction among young to middle-aged men (such as military service and family responsibilities) and middle-aged to elderly women (such as empty nest syndrome and aging) [60]. Rather than leaving these challenges to individuals alone, it is imperative to develop national-level solutions that provide institutional support and necessary psychosocial interventions tailored to each stage of the life cycle [60,67].

Some limitations of this study warrant discussion. First and foremost, this research focused only on life satisfaction, which is the cognitive component of subjective well-being. The study omitted the consideration of other aspects of mental well-being, including emotional, psychological, and social well-being, as described by [68]. This omission represents a potential limitation in comprehensively capturing the multifaceted nature of mental well-being. Second, a notable limitation that deserves mention is the fact that the vast majority of the variables studied were assessed using only one item. While enabling a streamlined data collection process suitable for national studies, short scales inadvertently introduce a significant limitation to the methodological rigor of the study. The inherent complexity and multidimensionality of the constructs require nuanced and comprehensive assessment methods to effectively capture their intricacies. Therefore, future studies should replicate these findings using longer and more reliable measures. An additional limitation arose from our reliance on a secondary data source, which limited our autonomy in selecting variables for inclusion in the study. Our efforts were constrained by the imperative to use only variables that were accessible within the confines of the Gallup data set. Finally, the cross-sectional nature of this research is a significant limitation, as it does not account for the potential temporal dynamics inherent in individual development and socio-environmental fluctuations. The absence of a longitudinal perspective prevents us from gaining insights into the evolving changes in life satisfaction over time, thereby highlighting the study’s limited scope.

Despite these acknowledged limitations, it is important to emphasize the merits of this study in providing valuable insights and guiding policy formulation based on a large national sample. By utilizing a comprehensive data set, the study effectively delves into the various aspects that contribute to the current state of life satisfaction among the Korean population.

## 5. Conclusions and Recommendations

In the Korean context, life satisfaction is better understood by considering age groups rather than focusing solely on gender. In particular, satisfaction with the standard of living, household income, and positive affect were key variables that could explain Koreans’ life satisfaction. In the context of the lower- and middle-income classes, annual household income is even more important than in the higher-income classes. In addition, considering factors such as reference income and subjective satisfaction, including satisfaction with living standards and household income, becomes crucial in determining overall life satisfaction. Consequently, policymakers have a responsibility to provide tangible support to ensure the well-being of individuals in the low-income brackets. This involves designing a set of social policies aimed at reducing income disparities and finding ways to increase overall happiness.

In addition, the strategy should include tailored interventions specifically designed for different subgroups based on gender, age, and income quintile. The goal is to increase life satisfaction. For example, it is imperative to provide job opportunities and social services specifically targeted to individuals in low-income groups to increase life satisfaction. Focusing on middle and late adulthood, periods characterized by gender and income inequalities, social support emerges as a key factor capable of improving overall quality of life. Similarly, tailored psychosocial interventions are critical for young and middle-aged men and older women who experience low levels of life satisfaction. Addressing these challenges requires a combination of personalized psychological interventions and targeted social policies.

## Figures and Tables

**Table 1:** The items used in the study.

Variable	Item Used	Response Format
Life satisfaction	Please imagine a ladder with steps numbered from zero at the bottom to ten at the top. The top of the ladder represents the best possible life for you and the bottom of the ladder represents the worst possible life for you. On which step of the ladder would you say you personally feel you stand at this time?	00 Worst possible to 10 best possible
Enjoyment	Did you experience the following feelings during a lot of the day yesterday? How about Enjoyment?	1 Yes
		2 No
Worry	Did you experience the following feelings during a lot of the day yesterday? How about Worry?	1 Yes
		2 No
Sadness	Did you experience the following feelings during a lot of the day yesterday? How about Sadness?	1 Yes
		2 No
Stress	Did you experience the following feelings during a lot of the day yesterday? How about Stress?	1 Yes
		2 No
Anger	Did you experience the following feelings during a lot of the day yesterday? How about Anger?	1 Yes
		2 No
Laughter	Did you smile or laugh a lot yesterday?	1 Yes
		2 No
Freedom	In (this country), are you satisfied or dissatisfied with your freedom to choose what you do with your life?	1 Satisfied
		2 Dissatisfied
Safe at night	Do you feel safe walking alone at night in the city or area where you live?	1 Yes
		2 No
Respect	Were you treated with respect all day yesterday?	1 Yes
		2 No
Learned	Did you learn or do something interesting yesterday?	1 Yes
		2 No
Satisfaction with city	Are you satisfied or dissatisfied with the city or area where you live?	1 Satisfied
		2 Dissatisfied
Household income satisfaction	Which one of these phrases comes closest to your own feelings about your household’s income these days?	1 Living comfortably on present income
		2 Getting by on present income
		3 Finding it difficult on present income
		4 Finding it very difficult on present income
Health problems	Do you have any health problems that prevent you from doing any of the things people your age normally can do?	1 Yes
		2 No
Social support	If you were in trouble, do you have relatives or friends you can count on to help you whenever you need them, or not?	1 Yes
		2 No
Satisfaction with standards of living	Are you satisfied or dissatisfied with your standard of living, all the things you can buy and do?	1 Satisfied
		2 Dissatisfied
Donated	Have you done any of the following in the past month? Donated money to a charity.	1 Yes
		2 No
Volunteered	Have you done any of the following in the past month? Volunteered your time to an organization	1 Yes
		2 No
Helped	Have you done any of the following in the past month? Helped a stranger or someone you didn’t know who needed help	1 Yes
		2 No
Corruption in business	Is corruption widespread within businesses located in Korea, or not?	1 Yes
		2 No
Corruption in government	Is corruption widespread throughout the government in Korea, or not?	1 Yes
		2 No

Note. All items had also two other response options: “don’t know” and “refuse to answer”. For the outcome and demographic variables, cases where respondents answered “don’t know” or “refused” were treated as missing data. The remaining variables were dummy coded, assigning a value of 1 for responses indicating “yes” or “satisfied” and 0 for responses indicating “no”, “dissatisfied”, “don’t know”, or “refused”.

**Table 2:** ANOVA results predicting life satisfaction.

		M	SD	N
Employment	Employed full time for an employer	6.007	1.936	2952
*df =* 5, 10,803 *F* = 17.655 *p* < 0.001 *η*^2^ = 0.008	Employed full time for self	5.660	2.242	1256
	Employed part-time do not want full time	6.317	2.064	794
	Unemployed	5.385	2.324	405
	Employed part-time want full time	5.539	2.378	406
	Out of workforce	5.956	2.255	4990
	Total	5.925	2.173	10,803
Education	Elementary	4.908	2.796	1429
*df =* 2, 13,816 *F* = 320.037 *p* < 0.001 *η*^2^ = 0.044	Secondary	5.701	2.112	7478
	Tertiary (four years beyond high school)	6.389	1.869	4912
	Total	5.864	2.161	13,819
Location	Rural or farm	5.231	2.399	1366
*df =* 3, 13,855 *F* = 48.936 *p* < 0.001 *η*^2^ = 0.010	Small town or village	5.746	2.136	1767
	Large city	5.954	2.119	6905
	Suburb of a large city	5.978	2.116	3821
	Total	5.863	2.161	13,859
Religious affiliation	Christian	6.142	2.162	4213
*df =* 3, 11,793 *F* = 30.909 *p* < 0.001 *η*^2^ = 0.008	Buddhist	5.661	2.240	2184
	Secular/non-religious	5.802	2.104	5073
	Other	5.725	2.253	327
	Total	5.895	2.163	11,797
Relationship status	Single	6.082	1.968	4164
*df =* 2, 13,484 *F* = 100.714 *p* < 0.001 *η*^2^ = 0.015	Married	5.909	2.114	8572
	Widow	4.895	2.742	751
	Total	5.907	2.126	13,487

**Table 3:** Comprehensive regression analysis.

	*B*	95.0% CI for *B*		*t*	*p*	Beta	Semi-Partial Correlation
		Low	Up				
(Constant)	2.341	2.034	2.649	14.920	0.000	-	-
Female	0.366	0.270	0.462	7.502	0.000	0.085	0.076
Age	−0.008	−0.011	−0.005	−5.782	0.000	−0.072	−0.059
Age-squared	0.000	0.000	0.000	0.949	0.342	0.011	0.010
Positive affect	0.341	0.213	0.468	5.233	0.000	0.066	0.053
Negative affect	−0.658	−0.819	−0.497	−8.030	0.000	−0.093	−0.081
Social support	0.362	0.249	0.474	6.301	0.000	0.071	0.064
Helped	0.124	0.031	0.216	2.622	0.009	0.028	0.027
Volunteer	0.218	0.109	0.328	3.910	0.000	0.042	0.040
Donated	0.096	−0.001	0.193	1.937	0.053	0.021	0.020
Freedom	0.230	0.135	0.324	4.771	0.000	0.052	0.048
Safe at night	−0.029	−0.124	0.065	−0.608	0.543	−0.007	−0.006
Respect	0.153	0.050	0.257	2.894	0.004	0.034	0.029
Learning experience	0.168	0.072	0.264	3.422	0.001	0.038	0.035
Health problems	−0.121	−0.230	−0.013	−2.198	0.028	−0.024	−0.022
Satisfaction with city	0.185	0.075	0.295	3.307	0.001	0.036	0.033
Corruption	−0.229	−0.343	−0.114	−3.919	0.000	−0.041	−0.040
Satisfaction w. stand. Of living	0.956	0.846	1.066	17.032	0.000	0.206	0.172
HH income satisfaction	0.477	0.410	0.545	13.832	0.000	0.163	0.140
Education level	0.292	0.216	0.368	7.548	0.000	0.085	0.076
Rural	−0.117	−0.272	0.037	−1.489	0.137	−0.016	−0.015
Small town or village	−0.104	−0.252	0.045	−1.372	0.170	−0.014	−0.014
Widow	−0.333	−0.532	−0.135	−3.288	0.001	−0.037	−0.033
Work full time	0.075	−0.056	0.205	1.120	0.263	0.015	0.011
Part-time, not looking for full time	0.175	−0.008	0.358	1.877	0.061	0.022	0.019
Out of workforce	0.044	−0.080	0.168	0.695	0.487	0.010	0.007
Christian	0.121	0.020	0.221	2.359	0.018	0.027	0.024
Buddhist	−0.066	−0.188	0.057	−1.052	0.293	−0.012	−0.011

**Table 4:** Unstandardized regression coefficients for age and gender groups.

	Gender		Age			
	Male	Female	15–24	25–44	45–64	65+
(Constant)	2.239 ***	2.776 ***	3.642 ***	3.480 ***	1.575 ***	1.908 ***
Female	-	-	0.129	0.441 ***	0.470 ***	0.196
Age	−0.009 ***	−0.008 ***	-	-	-	-
Age-squared	0.000 **	0.000	-	-	-	-
Positive affect	0.364 ***	0.316 **	0.419 **	0.322 **	0.405 ***	0.188
Negative affect	−0.664 ***	−0.628 ***	−0.646 ***	−0.986 ***	−0.364 *	−0.610 **
Social support	0.257 **	0.477 ***	0.252	0.208	0.398 ***	0.380 **
Helped	0.086	0.162 *	0.160	−0.010	0.197 *	0.118
Volunteer	0.171 *	0.267 ***	0.277 *	0.367 ***	0.245 **	0.026
Donated	0.080	0.104	0.134	0.042	0.019	0.242
Freedom	0.219 **	0.238 ***	0.361 ***	0.308 ***	0.215 *	0.036
Safe at night	0.012	−0.068	−0.110	−0.035	0.085	−0.084
Respect	0.096	0.212 **	0.184	0.088	0.099	0.242 *
Learning experience	0.157 *	0.182 **	0.369 ***	0.149	0.068	0.215
Health problems	−0.143	−0.101	−0.046	−0.224 *	−0.154	−0.047
Satisfaction with city	0.139	0.229 **	0.193	0.092	0.214 *	0.252
Corruption	−0.219 **	−0.247 **	−0.285 *	−0.231 *	−0.151	−0.296 *
Satisfaction w. stand. Of life	0.926 ***	0.986 ***	0.812 ***	0.901 ***	0.916 ***	1.229 ***
HH income satisfaction	0.512 ***	0.435 ***	0.345 ***	0.342 ***	0.586 ***	0.551 ***
Education level	0.334 ***	0.248 ***	0.112	0.140	0.382 ***	0.363 ***
Rural	−0.134	−0.117	−0.465 *	−0.240	−0.166	0.088
Small town or village	−0.075	−0.120	−0.182	0.003	−0.139	−0.193
Widow	−0.552 **	−0.196	-	-	-	-
Work full time	0.071	0.107	0.100	0.110	0.128	−0.168
Happy part-time, not looking for full time	0.205	0.141	−0.074	0.091	0.309 *	−0.008
Out of workforce	−0.070	0.113	−0.115	0.266 *	0.049	−0.117
Christian	0.152 *	0.083	0.092	0.304 ***	0.014	0.056
Buddhist	−0.005	−0.132	−0.235	0.170	−0.246 *	−0.035

* *p* < 0.05; ** *p* < 0.01; *** *p* < 0.001.

**Table 5:** Regression results across age and gender and income quintile groups.

	*R* ^2^	*F*	*df*	Most Important Predictors
Male	0.291	53.052 ***	26, 3356	SWSL, HH income, positive, education, and social support
Female	0.310	58.290 ***	26, 3374	SWSL, HH income, positive, education, and social support
15–24	0.226	13.865 ***	24, 1139	SWSL, positive, HH income, freedom, and learning experience
25–44	0.317	34.337 ***	24, 1779	SWSL, positive, HH income, negative, and female
45–64	0.331	45.220 ***	24, 2198	SWSL, positive, HH income, social support, and education
65+	0.286	26.253 ***	24, 1574	SWSL, positive, HH income, social support, and education
Poorest 20%	0.274	19.728 ***	26, 1356	SWSL, HH income, positive, age, and social support
Second 20%	0.299	21.083 ***	26, 1287	SWSL, social support, positive, education, and HH income
Middle 20%	0.298	20.367 ***	26, 1248	SWSL, positive, HH income, negative, and age
Fourth 20%	0.242	16.214 ***	26, 1324	SWSL, HH income, positive, respect, and helped
Richest 20%	0.245	18.006 ***	26, 1440	SWSL, HH income, positive, negative, and city satisfaction

Note. The estimates come from regression analyses using the method of enter. The important predictors come from separate regression analyses using the stepwise method. SWSL = satisfaction with standards of living; HH income = satisfaction with household income; positive = positive affect; negative = negative affect. *** *p* < 0.001.

**Table 6:** Income level predicting life satisfaction.

	M	SD	N
1 Poorest 20%	5.41	2.83	1880
2 Second 20%	6.37	2.39	1991
3 Middle 20%	6.68	2.18	1991
4 Fourth 20%	6.97	2.04	2064
5 Richest 20%	7.30	2.00	2273
Total	6.58	2.38	10,199

**Table 7:** Unstandardized regression coefficients for household income quintiles.

Predictor	1st	2nd	3rd	4th	5th
(Constant)	1.995 ***	2.222 ***	3.156 ***	3.111 ***	2.778 ***
Female	0.151	0.554 ***	0.301 **	0.321 **	0.337 **
Age	−0.020 ***	−0.008 **	−0.006 *	−0.004	−0.003
Age-squared	0.000 **	0.000	0.000	0.000	0.000
Positive affect	0.563 **	0.274	0.358 *	0.259 *	0.290 *
Negative affect	−0.398 *	−0.833 ***	−0.813 ***	−0.583 **	−0.702 ***
Social support	0.412 **	0.519 ***	0.240	0.113	0.333 *
Helped	−0.044	0.100	0.169	0.301 **	0.093
Volunteer	0.392 *	0.264 *	0.116	0.148	0.139
Donated	0.392 **	−0.084	−0.068	−0.006	0.194 *
Freedom	0.223 *	0.110	0.182	0.268 **	0.321 **
Safe at night	0.071	−0.111	−0.268 **	0.074	0.095
Respect	0.134	0.164	0.132	0.285 **	−0.048
Learned	−0.084	0.344 **	0.029	0.198 *	0.248 *
Health problems	0.082	−0.221	−0.309 *	−0.142	−0.048
Satisfaction with city	0.323 *	−0.022	0.292 *	0.003	0.338 **
Corruption	−0.282	−0.252	−0.276 *	−0.268 *	−0.057
Satisfaction w. stand. Of life	0.991 ***	0.803 ***	0.988 ***	0.915 ***	1.023 ***
HH income satisfaction	0.436 ***	0.370 ***	0.461 ***	0.484 ***	0.427 ***
Education level	0.353 **	0.441 ***	0.158	0.131	0.092
Rural	0.157	0.037	−0.296	−0.297	−0.352
Small town or village	−0.130	0.108	−0.211	−0.117	−0.131
Work full time	−0.129	0.366 *	0.083	−0.075	0.174
Happy part-time, not looking for full time	−0.180	0.500 *	0.210	−0.069	0.484 *
Out of workforce	−0.198	0.257	0.113	0.136	0.120
Christian	0.112	0.230 *	0.061	0.017	0.161
Buddhist	−0.133	0.104	−0.287 *	−0.046	−0.019

* *p* < 0.05; ** *p* < 0.01; *** *p* < 0.001.

## Data Availability

This study used data from the Gallup World Poll. The data are available from Gallup (for more information, see https://www.gallup.com/home.aspx, accessed on 1 July 2023).
